# Long-Range Regulatory Synergy Is Required to Allow Control of the TAC1 Locus by MEK/ERK Signalling in Sensory Neurones

**DOI:** 10.1159/000322010

**Published:** 2010-12-16

**Authors:** Lynne Shanley, Scott Davidson, Marissa Lear, Anil Kumar Thotakura, Iain Joseph McEwan, Ruth A. Ross, Alasdair MacKenzie

**Affiliations:** ^a^School of Medical Sciences, University of Aberdeen, Aberdeen, UK; ^b^Immunology Department, Imperial College London, Hammersmith Hospital, London, UK

**Keywords:** Substance-P, TAC1 gene, Gene regulation, Regulatory synergy, Enhancer-promoter selectivity, MEK/ERK signalling, Comparative genomics, Sensory neurons, Capsaicin, Hyperalgesia

## Abstract

Changes in the expression of the neuropeptide substance P (SP) in different populations of sensory neurones are associated with the progression of chronic inflammatory disease. Thus, understanding the genomic and cellular mechanisms driving the expression of the TAC1 gene, which encodes SP, in sensory neurones is essential to understanding its role in inflammatory disease. We used a novel combination of computational genomics, primary-cell culture and mouse transgenics to determine the genomic and cellular mechanisms that control the expression of TAC1 in sensory neurones. Intriguingly, we demonstrated that the promoter of the TAC1 gene must act in synergy with a remote enhancer, identified using comparative genomics, to respond to MAPK signalling that modulates the expression of TAC1 in sensory neurones. We also reveal that noxious stimulation of sensory neurones triggers this synergy in larger diameter sensory neurones – an expression of SP associated with hyperalgesia. This noxious stimulation of TAC1 enhancer-promotor synergy could be strongly blocked by antagonism of the MEK pathway. This study provides a unique insight into the role of long-range enhancer-promoter synergy and selectivity in the tissue-specific response of promoters to specific signal transduction pathways and suggests a possible new avenue for the development of novel anti-inflammatory therapies.

## Introduction

Understanding how the human genome regulates the changes in tissue-specific gene expression that contribute to the progression of disease is critical to understanding the disease process [1]. For example, neurogenic inflammation is a critical component of inflammatory disease [2]. The neuropeptide substance P (SP), encoded by the TAC1 gene, acts as a key regulator of neurogenic inflammation [3,4,5]. Consistent with this role, SP is usually expressed in nociceptive C-fibre sensory neurones within the dorsal root ganglia (DRG) [5]. In addition, it has been established that the expression of SP is upregulated in larger diameter A- and B-fibre sensory neurones following noxious stimulation, an expression pattern also associated with the development of inflammatory pain and hyperalgesia [6,7]. Thus, identifying the genomic sequences that control TAC1 expression and understanding how their activity is controlled by signal transduction systems is critical to understanding the processes governing neurogenic inflammation.

In addition to TAC1, noxious stimulation and nerve injury upregulates the activity of a number of signal transduction pathways in DRG neurones [8,9]. Recently, the roles of the MAPK pathways, which include the MEK/ERK pathway, have come under particular scrutiny because of their known role in inflammatory pain and as potential therapeutic targets [8,10,11,12]. For example the MEK/ERK pathway is activated in sensory neurones following nerve damage/noxious stimulation, and the application of specific antagonists, such as PD98059, has been shown to significantly reverse induced hyperalgesia [8,13]. Furthermore, MEK/ERK pathways are activated in populations of larger DRG neurones following nerve damage or noxious stimulation [14,15,16,17,18,19].

Considering their parallel roles in the production of neurogenic inflammation and the maintenance of hyperalgesia, we explored the possibility that MAPK pathways could play a role in the regulation of the TAC1 gene in sensory neurones. We demonstrated that activation of MAPK pathways induced the expression of TAC1 in sensory neurones, but could not activate the TAC1 promoter. We used comparative genomics to identify and isolate a highly conserved and remote candidate regulatory element that lay 214 kb 5′ of the TAC1 locus which we called ECR2 (evolutionary conserved region 2). We observed that in the presence of ECR2, the TAC1 promoter could respond to MAPK signalling in sensory neurones, demonstrating a previously unknown requirement for synergy between a remote enhancer region and a gene promoter in response to MAPK signalling. Interestingly, noxious stimulation of sensory neurones also induced the activity of this synergy in larger diameter neurones consistent with the possible role of ECR2-TAC1 promoter synergy in hyperalgesia – a chronic condition that is often associated with inflammatory disease. Significantly, antagonism of the MEK/ERK pathway strongly reversed the effects both of angiotensin and capsaicin. The mechanistic linkage of two important regulators of neurogenic inflammation, the MEK/ERK pathway and the TAC1 gene, using comparative genomics indicates a future direction for understanding the interactions of the signal transduction systems and genomic sequences involved in modulating disease-related genes.

## Materials and Methods

### Bioinformatic Analysis

**Fig. 1:**
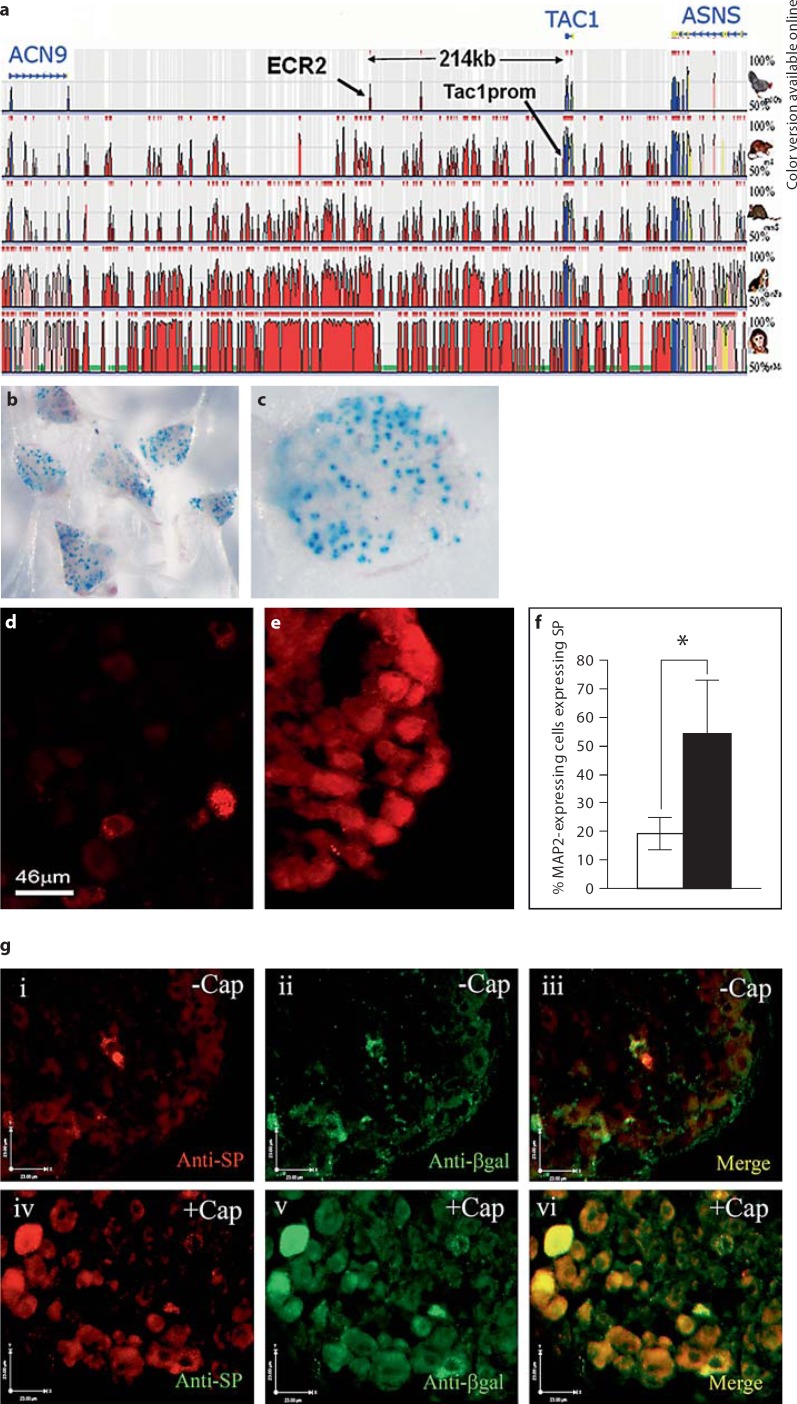
**a** Plot from the ECR Browser comparing 850 kb surrounding the human *TAC1* locus with (from top to bottom) chicken, rat, mouse, dog and rhesus monkey genomes. The VISTA plots represent the genomic extent of (from left to right) the coding regions for ACN9 (homolog of yeast acetate non-utilizing gene 9, involved in gluconeogenesis), TAC1 (tachykinin 1) and ASNS (asparagine synthetase). The xaxis represents linear distance with reference to the human genome sequence. The y-axis represents levels of sequence conservation between 50 and 100%. Blue lines with chevrons represent the genomic extent of each gene. Red, blue, pink and yellow peaks represent areas of sequence conservation (>75% over 100 bp) in intergenic non-coding, exonic, intronic and untranslated regions, respectively (colors in online version only). **b, c** Whole mount X-galstained DRG preparations from neonate mice transgenic for the ECR2-TAC1prom-LacZ transgene. **d, e** Florescent immunohistochemical analysis using an anti-SP antibody showing expression in whole mouse neonate DRG neurones after 24 h exposure to vehicle control (**d**) or 10 μM capsaicin (**e**). **f** Bar graph representing the combined results of 3 different experiments on different groups of animals at different times (n = 3) showing proportions of MAP2-expressing cells in DRG neurons that also express SP in the absence (white bar) or presence (black bar) of capsaicin. **g i–vi** Fluorescence images of an immunohistochemical study of SP and transgene expression on whole DRG explant cultures derived from ECR2-TAC1prom-LacZ transgenic neonates. Cultures represented by **i, ii** and **iii** were treated with vehicle and cultures represented by **iv, v** and **vi** were treated for 24 h with capsaicin prior to fixing and immunohistochemical analysis. Immunohistochemical analysis was carried out using anti-SP (**i** and **iv**) or anti-β-gal (**ii** and **v**) as primary antibodies. **iii** and **vi** represent merged images where co-localisation is in yellow. White arrows indicate 23 μm.

Comparative genomic analysis was carried out using the ECR browser (http://ecrbrowser.dcode.org/) [20] and the UCSC browsers (http://genome.ucsc.edu/) (see fig. 1a). Transcription factor binding sites were predicted using Match (http://www.gene-regulation.com/cgi-bin/pub/programs/match/bin/match.cgi?).

### Generation of Plasmid Constructs (see fig. 2b and c)

*pMinprom-Luc*. pGL4.23 (Promega) was renamed pMinprom-Luc for the purposes of clarity.

*pTAC1prom-Luc*. A *Nco*I/*Bgl*II fragment was isolated from pGEMTAC1prom (see below) and cloned into the *Nco*I/*Bam*HI site of pGL4.23 (Promega).

*pECR2minprom-Luc*. A *Zra*I/*Sac*I fragment of pGEMhECR2 (see below) was ligated into the *Eco*RV/*Sac*I site of pGL4.23.

*pECR2TAC1prom-Luc*. A *Zra*I/*Sac*I fragment of pGEM5hECR2 was ligated into the *Eco*RV/*Sac*I site of pTAC1prom-Luc.

*ph*β*gprom-LacZ*. This plasmid was provided as a kind gift from Robert Kraumlauf and is a derivative of a plasmid known as BGZ40 and was renamed for clarity.

**Fig. 2:**
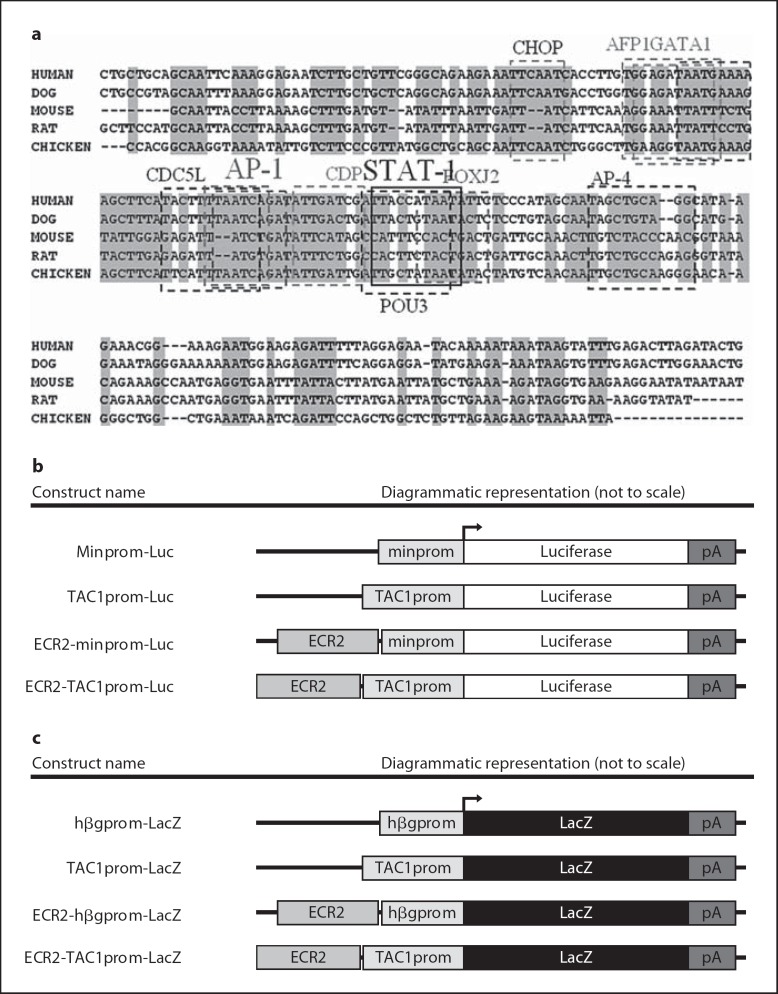
**a** Sequence alignment of 240 bp of the most highly conserved region of ECR2 highlighting the presence of several conserved transcription factor binding sequences as predicted using the TRANSFAC database. Transcription factor consensus sequences have been highlighted using broken boxes. Sequences conserved back to chicken are highlighted in filled grey boxes. **b, c** Diagrammatic representation (not to scale) demonstrating the linear relationships of the components of the different luciferase (**b**) and LacZ constructs (**c**) used in the current study. pA = SV40 polyadenylation sequence; LacZ = β-galactosidase; hβgprom = human β-globin promoter; TAC1prom = TAC1 promoter; minprom = minimal promoter (Promega).

*pTAC1prom-LacZ*. Comparative genomic analysis between rodents and humans of the TAC1 gene was used to identify a 960-bp highly conserved region of the TAC1 proximal promoter (fig. 1a; chr7: 20594284–20595244). This sequence was amplified from human placental DNA using high-fidelity PCR (Expand HiFi system, Roche) using the following oligonucleotide sequences: hTAC1prom.for for CCT TTA GTG ACA AGG GTG AGG, and hTAC1prom.rev for CAC TTA CTG CGA CGG ACA GT. PCR products were blunt-end-cloned into pGEM5z (Promega) to form pGEMTAC1prom and sequenced (ABI 377 sequencer) to determine amplification fidelity. The pSBLacZ expression vector (a kind gift from Sanbing Shen) is based on a fusion of pBluescript and a LacZ reporter gene containing an *Nco*I site at its transcriptional start site. A new multiple cloning site was created within this plasmid by the ligation of the following oligonucleotides to produce pMarI: Al1.for for 5′-TTA CTA GTG CATGCT ACT GCA GGG ATC CTA CCG CGG CCA TGG TT-3′, and Al1a.rev for 5′-AAC CAT GGC CGC GGT AGG ATC CCT GCA GTA GCA TGC ACT AGT AA-3′. The human TAC1 promoter from pGEMTAC1prom was cloned into a *Bam*H1 and *Sac*II site of pMarI to form pTAC1prom-LacZ (fig. 2c). For the production of transgenic lines, this construct was linearised using *Not*I and *Apa*I digestion and gel-purified for pronuclear microinjection.

*pECR2-h*β*gprom-LacZ*. The ECR2 sequence used in this study was isolated from human placental DNA using high-fidelity PCR (Expand HiFi system, Roche) using the following oligonucleotide primers: ECR2_FOR for TTT TGG GAG AAT GGA AGT GG, and ECR2_REV for TGG CTT GGG GTA ATC TTT TT (MWG). The 1,000-bp PCR product was digested with *Eco*RV and *Sac*I to give a 900-bp fragment that was cloned into equivalent sites within pGEM5z (Promega) to form pGEM5hECR2 and sequenced (ABI 377 sequencer) to determine amplification fidelity. To produce pECR2-hβg-LacZ, an 889-bp ECR2 fragment was digested out of pGEM5hECR2 using *Eco*RV and *Apa*I and ligated into the *Apa*I and *Hin*dIII (blunt-ended using Klenow) of the β-galactosidase (LacZ) reporter construct phβgprom-LacZ (fig. 2c).

*pECR2-TAC1prom-LacZ*. To produce pECR2-TAC1prom-LacZ, ECR2 was recovered from pGEMECR2 by digestion with *Nsi*I (blunt-ended with Klenow) and *Sph*I and ligated into pTAC1prom-LacZ digested with *Sph*I/*Bam*H1 (blunt-ended) to form pECR2-TAC1prom-LacZ (fig. 2c). For the production of transgenic lines, this construct was linearised using *Not*I and *Apa*I digestion and gel-purified for pronuclear microinjection.

### Transgenic analysis

Transgenic lines were produced by pronuclear microinjection as previously described [21,22].

### Primary Cell Culture and Transfection

Neonatal rat DRG were cultured as described previously [23]. For luciferase studies, cells were transfected using magnetic particles (NeuroMag, Oz Biosciences) following the manufacturer's instructions. For LacZ cell-counting studies, cells were Amaxa-transfected according to the manufacturer's instructions using the program G-013 (Amaxa). The transfected cell suspension was transferred into a poly-*L*-lysine/laminin-coated 24-well culture dish containing 0.3 ml of culture media supplemented with NGF (0.1 μg/ml). Cells were exposed to treatments for 12 h. Unless otherwise stated, the vehicle control for all experiments was water.

### Dual Luciferase Assays

For luciferase analysis, primary DRG neurones cells were cultured and transfected as described above. Firefly luciferase assays were internally controlled using a co-transfected renilla luciferase control plasmid. Cell extracts and dual luciferase assays were carried out as per the manufacturer's instructions (Promega) and assayed in a GloMax 96 Microplate Luminometer with dual injectors (Promega).

### X-Gal Staining and Cell Counting

Following transfection with DNA, DRG neurons were allowed to adhere to poly-*L*-lysine/laminin-coated glass cover slips and were cultured in a solution containing 10 μ*M* capsaicin, 10 μ*M* angiotensin or vehicle (DMSO). Cultures were left at 37°C for 24 h before the culture media was removed, and cells were fixed with 4% paraformaldahyde. Expression of the LacZ was visualised by staining with X-gal stain for 2 h as previously described [21,22]. The number of blue DRG neurons as a percentage of the total number of neurons was assessed by cell counting on an inverted DIC microscope. In order to minimise the effects of variation between different groups of animals, a CMV reporter construct was transfected at the same time to normalise transfection efficiencies.

### Transgenic DRG Explant Analysis and Immunocytochemistry

Whole DRG explants were dissected from transgenic neonates and placed in the same culture conditions as described above. These explants were then treated with DMSO or capsaicin (10 μ*M*) for 24 h, fixed in 4% paraformaldehyde and incubated with 30% sucrose in optimal cutting temperature media overnight. 10-μm sections were permeabilised with 0.1% SDS for 5 min, and then incubated in 10% foetal calf serum in Tris-buffered saline with 1% triton for 10 min. Sections were washed 3 times for 5 min in Tris-buffered saline with 1% triton and treated sequentially in primary antibodies overnight (rabbit-anti-β-gal, 1:200, rat-anti-SP, AbCam). Antibodies were visualised by incubation with the appropriate secondary antibody (diluted to 1:250) for 40 min at room temperature (goat-anti-rat Texas red, donkey-anti-rabbit ALEXA 488 or donkey-anti-goat ALEXA 488, all from Molecular Probes). Observations and analyses of cell numbers expressing specific antigens (SP or β-gal) were undertaken on a minimum of 3 separate occasions from DRG derived from animals from 3 different litters (n = 3). On any given day, treated and untreated sections were subjected to immunohistochemistry on the same slides and photographed with a fluorescent microscope under identical levels of illumination. Cell measurements were taken across the widest part of each cell as previously described [24].

### Quantitative RT-PCR

DRG explants were cultured for 12 h in the presence of angiotensin as described above. RNA was extracted using TRIzol (Invitrogen) and 1 μg of each RNA sample was DNase I-treated with 1 U of amplification grade DNase I (Invitrogen) following the manufacturer's protocol. Subsequently, 1 μl of 5 ng/μl oligo dT (Promega) was added to each sample and heated for 10 min at 70°C. First-strand synthesis was then carried out using Superscript II reverse transcriptase (Invitrogen) following the manufacturer's protocol to give total cDNA.

For TaqMan-based qRT-PCR, 1 μl of cDNA was used per qPCR reaction for 40 cycles using FAM-labelled TaqMan probes for TAC1 mRNA targets. All reactions contained a DIG-labelled probe set for mouse GAPDH as an internal control to normalize expression levels. Each reaction consisted of 10 μl Lightcycler 2.0 Probes Master mix, 1 μl TaqMan probe set (Applied Biosystems), 0.5 μl GAPDH TaqMan control probe set (Applied Biosystems), 1 μl cDNA and 7.5 μl nuclease-free water (Ambion). The TAC1 TaqMan probe set was a TaqMan Gene Expression Assay (Applied Biosystems).

### Image Capture and Analysis

Images were captured on Qicam monochrome camera mounted on a Nicon Eclipse 400 fluorescence microscope and processed using Improvision Velocity software v4.1. Figures were assembled with Adobe Photoshop CS3 software.

### Statistical Analysis

All experiments were repeated a minimum of 3 times on separate intervals using a minimum of 3 separate groups of animals (n ≥3). Where appropriate, Levene's tests were carried out on all data sets to test whether the data fulfilled the requirement for parametric testing. Where assumptions were met, the data were analysed using 2-tailed t tests. In cases where data sets did not meet the requirements for parametric testing, the data were analysed for significance using the Mann-Whitney test. Where sample sizes differed, the data were analysed using an unpaired t test.

## Results

### Expression of the TAC1 Gene Is Increased in DRG Neurones following Activation of MAPK Pathways

**Fig. 3:**
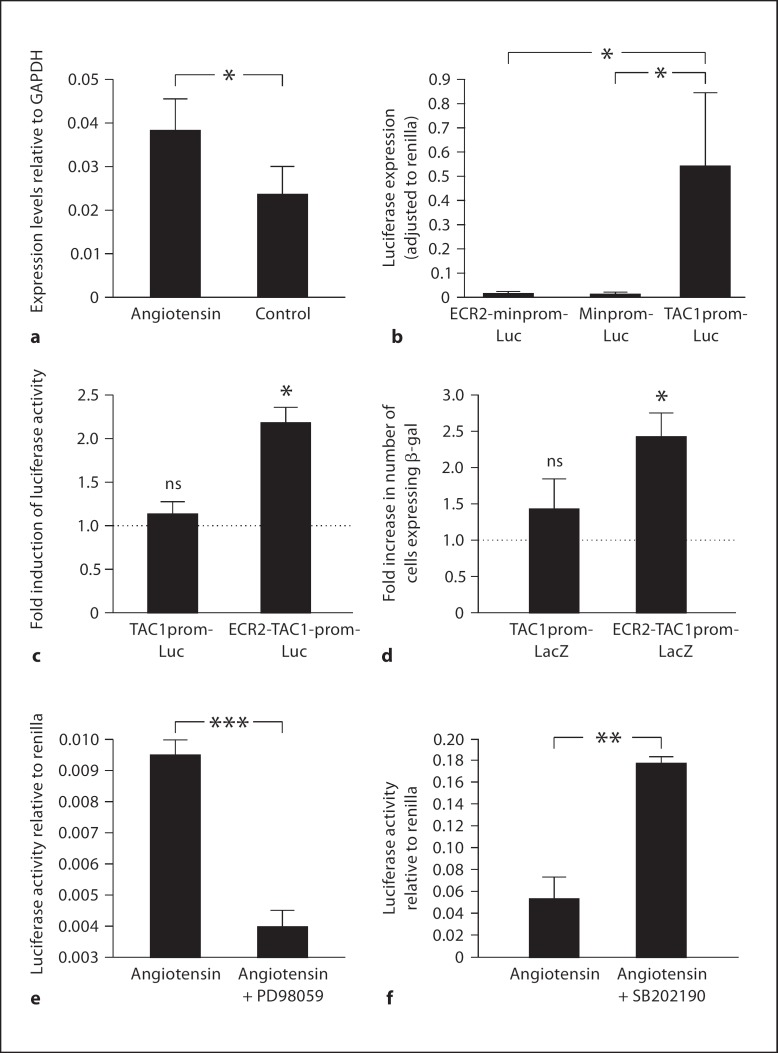
**a** rtPCR analysis of the levels of TAC1 mRNA derived from whole cultured DRG explants cultured in the presence of absence of the MAPK agonist angiotensin II (n = 3, * p < 0.05). The y-axis represents TAC1 mRNA abundance relative to GAPDH transcripts. **b** Luciferase reporter gene assay comparing the ability of different promoter constructs described in figure 2b to support luciferase expression in primary sensory neurones. The y-axis represents firefly luciferase activity adjusted to renilla luciferase activity (n = 3, * p < 0.05). **c** Fold change in the levels of luciferase expression (normalised against renilla luciferase) following angiotensin II treatment of primary DRG neurones transformed with either the TAC1prom-Luc or the ECR2-TAC1prom-Luc constructs (n = 3, * p < 0.05). **d** Fold change in the number of cells expressing the LacZ marker gene following treatment of primary DRG neurones transformed with either the TAC1prom-LacZ or the ECR2-TAC1prom-LacZ construct and treated with the MAPK agonist angiotensin II (n = 5, * p < 0.05). **e, f** Dual luciferase reporter studies carried out on primary sensory neurones following their transfection with the ECR2-TAC1prom-Luc construct and normalised using renilla luciferase demonstrating the effects of the MEK antagonist PD98058 (**e**) and the p38 antagonist SB202190 (**f**) on the ability of angiotensin II to influence the activity of the ECR2-TAC1prom-Luc (** p < 0.01; *** p < 0.005).

Neonate mouse DRG explants were cultured in the presence or absence of the MAPK agonist angiotensin II for 24 h (n = 4). RNA was then extracted from these explants and the levels of TAC1 expression were measured using real-time rtPCR. In this way we were able to demonstrate that levels of the expression of the TAC1 gene were increased significantly in DRG explants exposed to angiotensin II (fig. 3a). These results support the hypothesis of a mechanistic linkage between the activation of MAPK pathways with the expression of the TAC1 gene.

### Angiotensin II Does Not Affect the Activity of the TAC1 Promoter

The expression of all genes, including the TAC1 gene, relies on promoter elements that lie immediately 5′ of their transcriptional start sites. The TAC1 promoter lies between −865 to +92 of the TAC1 gene and can support marker gene expression when injected or virally infected into cultured DRG neurones [25,26,27]. In support of these previous studies we used comparative genomics to demonstrate that 966 bp immediately 5′ of the TAC1 +1 had been conserved (>75% over 100 bp) since the earliest common ancestor of rodents and humans (fig. 1a). We cloned this conserved region of the human TAC1 locus and made luciferase reporter plasmids in which the TAC1 promoter region was fused directly to the first codon (ATG) of the luciferase gene (fig. 2b). These constructs were transfected into primary DRG neurones that were then cultured for 16 h in the presence or absence of the MAPK agonist angiotensin II. TAC1prom-Luc was able to drive significantly higher levels of luciferase expression than the minprom-Luc vector in sensory neurones as previously reported (fig. 3b) [25,26,27]. However, we found no significant increase in the activity of the TAC1 promoter by luciferase assay after incubation in angiotensin II (fig. 3c). This data confirms previous findings that the TAC1 promoter sequence has an intrinsic ability to support gene expression in DRG-derived cells [25,26,27]. However, unlike the endogenous TAC1 gene, the TAC1prom sequence is unable to respond to angiotensin II in isolation.

### ECR2 Allows TAC1prom to Respond to Activation of MAPK Pathways in Primary DRG Neurones

Comparative genomics has previously been used for the identification of gene regulatory sequences that control the expression of genes [21,22,28,29,30,31,32]. We used comparative genomics to compare the human genome to that of the mouse, rat, dog and chicken genomes within 380 kb of the TAC1 locus. In this way we were able to identify a 240-bp region of homology (75% over 100 bp) that lay 214 kb 5′ of TAC1 (chr7: 96983940–96984126), which we call ECR2 (fig. 2a).

TRANSFAC analysis of this sequence predicted the highly conserved binding sites of a number of different transcription factors including those of the AP1 and STAT1 transcription factor proteins that are known to play an important role in the inflammatory response (fig. 2a). We cloned 1 kb of DNA surrounding the ECR2 sequence using high-fidelity PCR and used this sequence to produce a series of luciferase reporter constructs (fig. 2b). When combined with an exogenous promoter (minprom) ECR2 could not support reporter gene expression when transformed into primary DRG neurones (fig. 3b). However, when placed next to the TAC1prom sequence, we observed a significant increase in luciferase activity in the presence of angiotensin II (fig. 3c).

Because these primary neuronal cultures represented a heterogeneous population of phenotypically diverse cells, we wanted to determine whether the increase in gene expression observed in the presence of angiotensin was a result of an increase in the levels of gene expression in a fixed population of cells or an increase in the numbers of cells expressing the transgene. We subcloned different combinations of ECR2 and TAC1prom into a LacZ reporter construct and transfected these into dissociated DRG neurones (fig. 2c). We observed a significant increase in the numbers of DRG neurones transfected with the ECR2-TAC1prom-LacZ construct that expressed β-gal following incubation in the presence of angiotensin II (fig. 3d). However, no significant increase in the numbers of cells expressing β-gal was observed following transfection with LacZ reporter vectors containing TAC1prom in isolation or ECR2-hβg in the presence of angiotensin.

These results support the existence of regulatory synergy between the TAC1prom sequence and ECR2 in the cellular induction of TAC1 gene expression in DRG neurones following activation of MAPK pathways. In addition, our data suggests that much of the upregulation of TAC1prom activity observed through our luciferase assays may be due to an increase in the numbers of cells in which TAC1prom is active.

### Antagonism of the MEK/ERK Pathway Reduces the Activation of ECR2-TAC1prom by Angiotensin

The MAPKinases are divided into 3 distinct pathways: p38, JNK and MEK/ERK. In order to determine which of these pathways is responsible for the upregulation of ECR2-TAC1prom synergy by angiotensin, we transfected primary sensory neurones with the ECR2-TAC1prom-Luc construct and incubated them in the presence of angiotensin and antagonists of the JNK (SP600125), p38 (SB202190) and MEK/ERK (PD98059) pathways. The JNK kinase antagonist had no significant effect on the activation of ECR2-TAC1prom-Luc by angiotensin (data not shown). Only PD98059 was able to significantly reverse the effects of angiotensin on the ECR2-TAC1prom-Luc construct, suggesting that MAPK control of ECR2-TAC1prom is modulated by the MEK/ERK pathway (fig. 3e). In contrast, application of SB202190 actually increased activity of the transgene, suggesting that the p38MAPK pathway plays an antagonistic role against an alternative stimulatory signalling pathway (fig. 3f).

### ECR2 Works in Synergy with TAC1prom to Support Gene Expression within SP-Expressing DRG Neurones

Although we have shown that ECR2 and TAC1 work in synergy with each other to maintain TAC1prom activity in mixed populations of DRG neurones, we have not shown that ECR2 and TAC1prom can support gene expression in SP-expressing DRG neurones. In order to address this deficit, we used pronuclear microinjection to produce a series of mouse transgenic lines with the TAC1prom-LacZ, and ECR2-TAC1prom-LacZ constructs.

None of the 3 lines produced using TAC1prom-LacZ demonstrated detectible β-galactosidase expression in SP-expressing DRG neurones. These observations are consistent with previous studies [33,34] where it was concluded that, although active in transiently injected or transfected primary DRG neurones where the reporter plasmid remains extra-chromosomal, the TAC1 promoter tended to be silenced in transgenic lines. This may occur as a result of the transgene silencing by methylation during embryonic development.

However, of the 7 transgenic lines produced using the ECR2-TAC1prom-LacZ construct, 4 expressed β-galactosidase within subsets of SP-expressing DRG neurones (fig. 1b, c and g). We have shown that ECR2 allows the TAC1 promoter to respond to MAPK signalling that induces the expression of TAC1. These transgenic studies also demonstrate that ECR2 allows the TAC1 promoter to be active within SP-expressing sensory neurones. Together, these observations support the hypothesis that synergism between ECR2 and TAC1prom is responsible for the expression of the TAC1 gene in sensory neurones.

### Activation of TRPV1, a Key Integrator of Noxious Stimuli, Induces the Expression of SP in DRG Neurons

A number of studies have shown that TAC1 expression is increased in sensory neurones following noxious stimulation [6,7,35,36,37]. We examined the expression of SP in cultured DRG explants following exposure to the TRPV-1 agonist capsaicin, which is a well-characterised inflammatory pain paradigm and was previously shown to induce the expression of TAC1 and SP within sensory neurones [38,39]. In 3 different experiments carried out on 3 separate occasions with different groups of animals, we observed a consistent and significant upregulation of the numbers of cells expressing SP, a recognised marker of sensory neurones, within these explants compared to non-treated controls (fig. 1d–f). These observations support previous studies describing the stimulatory effects of capsaicin on TAC1 expression [38,39].

### The TAC1 Promoter Requires ECR2 to Respond to Capsaicin Treatment

**Fig. 4:**
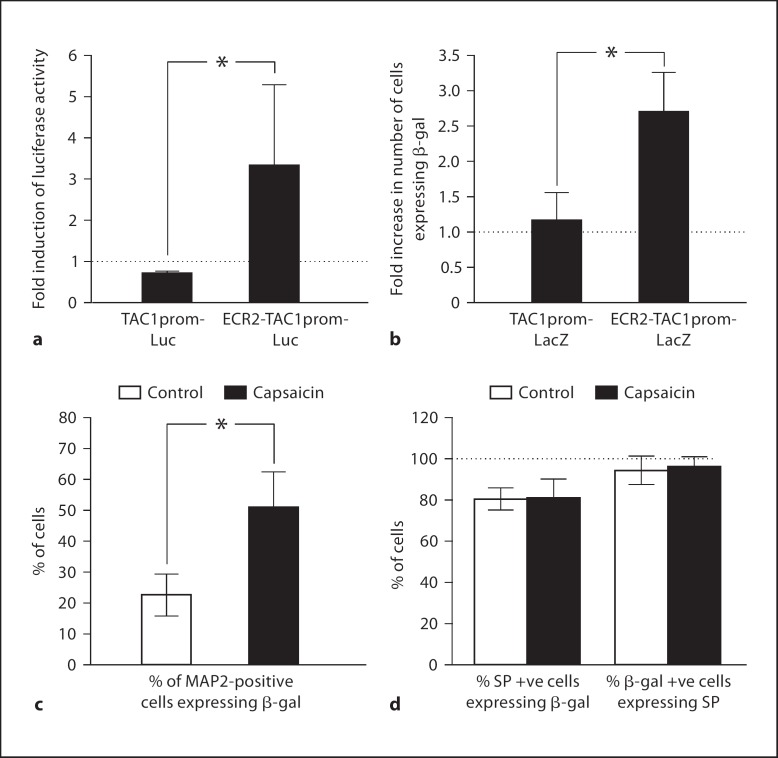
**a** Fold change in the levels of luciferase expression following capsaicin treatment of primary DRG neurones transformed with either the TAC1prom-Luc or the ECR2-TAC1prom-Luc constructs (n = 3, * p < 0.05). **b** Fold change in the number of cells expressing the LacZ marker gene following capsaicin treatment of primary DRG neurones transformed with either the TAC1prom-LacZ or the ECR2-TAC1prom-LacZ construct (n 1 3, * p < 0.05). **c** Graphical representation of the proportion of MAP-positive DRG neurones expressing β-gal in ECR2-TAC1prom-LacZ transgenic DRG explants in the absence (white bar) or presence (black bar) of capsaicin (n = 3, * p < 0.05). **d** Co-localisation of SP expression and ECR2-TAC1prom-LacZ transgene activity in whole transgenic DRG neurones in the presence (white bar) or absence (black bar) of capsaicin treatment (n = 3).

In order to determine whether the activation of the transgene by capsaicin was mediated by either the TAC1prom or ECR2 sequences, we transfected primary sensory neurones with the constructs described in figure 2b and c. In a similar manner to angiotensin, neither of the Minprom, hβgprom or ECR2 sequences supported any marker gene expression in sensory neurones in the presence or absence of capsaicin. However, we observed that TAC1prom could respond to capsaicin in the presence of ECR2, suggesting the presence of regulatory synergy in the induction of TAC1 by capsaicin (fig. 4a and b). These experiments demonstrate a parallel between the mechanisms controlling the induction of TAC1prom by angiotensin and capsaicin.

### Capsaicin Changes the Cell-Specific Expression of Both SP and the ECR2-TAC1prom Transgene in Transgenic DRG Explants

**Fig. 5:**
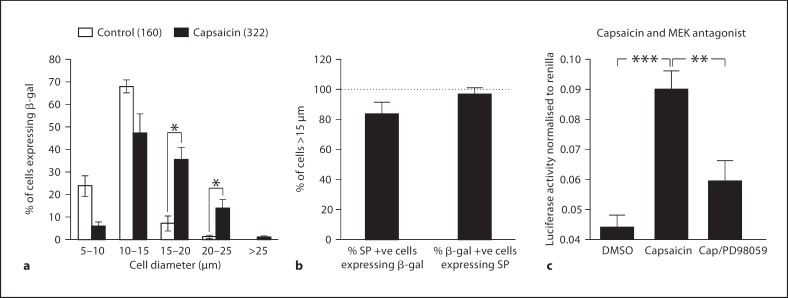
**a** The size distribution (diameter in μm) of neurones within ECR2-TAC1prom-LacZ transgenic neonate DRG explants analysing β-gal expression before and after capsaicin treatment (n = 3) showing a significant shift in the proportion of cells with a diameter greater than 15 μm expressing the transgene. Numbers in brackets represent the total numbers of β-gal expressing cells analysed (n = 3, * p < 0.05). **b** The proportion of cells of 15 μm in diameter or greater that co-express SP and β-gal following capsaicin treatment (n 1 3). **c** Dual luciferase reporter studies carried out on primary sensory neurones following their transfection with the ECR2-TAC1prom-Luc construct and normalised using renilla luciferase demonstrating the ability of the MEK antagonist PD98058 to reverse the effects of capsaicin (n = 3, ** p < 0.01, *** p < 0.005).

In order to observe the influence of capsaicin on the expression of both SP and the ECR2-TAC1prom-LacZ transgene in the different populations of neurones making up the DRG, we isolated whole DRG explants from transgenic neonates. These were cultured for 24 h in the presence or absence of capsaicin. Consistent with the experiments described above, subsequent immunohistochemical analysis using both anti-SP and anti-β-gal antisera demonstrated that the expression of SP and the ECR2-TAC1prom-LacZ transgene was observed in significantly more neurones following capsaicin treatment (n >3, fig. 1g iv–vi and 4c). Analysis of cellular co-expression of SP and β-gal demonstrated that the ECR2-TAC1prom-LacZ transgene was expressed within greater than 80% of SP expressing cells and virtually all cells that expressed the transgene also expressed SP (fig. 1g iv–vi and 4d). Analysis of the diameters of the cells co-expressing SP and the transgene following capsaicin induction showed that the numbers of neurones of a diameter of 15 μm or greater also increased significantly (n = 3, fig. 1g iv–vi and 5a). Intriguingly, virtually all cells of a diameter equal to or greater than 15 μm that expressed the transgene also expressed SP following capsaicin treatment (fig. 5b). These observations suggest that, by working in synergy, ECR2 and TAC1prom play an important role in the noxious induction of TAC1 expression in larger diameter neurones [6,7,35,36,37].

### Antagonism of the MEK/ERK Pathway Blocks the Effects of Capsaicin

We have shown that the TAC1prom sequence can only respond to either angiotensin or capsaicin in the presence of the ECR2 sequence. This suggests that there are common mechanisms controlling the effects of capsaicin and angiotensin on TAC1 promoter activity in sensory neurones through ECR2. In order to address this possibility we transfected primary DRG neurones with the ECR2-TAC1prom-Luc plasmid and cultured them in the presence of capsaicin or capsaicin plus the MEK/ERK antagonist PD98059. As before, we observed strong and significant upregulation of the activity of the ECR2-TAC1prom-Luc plasmid in these cells following exposure to capsaicin (fig. 5c). However, in the presence of PD98059, capsaicin was unable to induce significantly higher expression of the reporter construct (fig. 5c). These results confirm the hypothesis that the noxious induction of ECR2-TAC1prom by capsaicin is mediated through the MEK/ERK pathway.

## Discussion

Because of its critical role in neurogenic inflammation, the promoter of the TAC1 gene has been well characterised and has been shown to respond to a number of important inflammatory queues including PKA signalling and induction of the TrkA receptor by NGF [26,27,40,41,42,43,44,45,46]. However, despite their known roles in modulating inflammation and hyperalgesia, the role of the MAPK in influencing the activity of the TAC1 promoter has been relatively under-explored. Therefore, we explored the possibility of a mechanistic linkage between MAPK pathways and the expression of the TAC1 promoter within sensory neurones. We initially demonstrated the ability of angiotensin II to induce TAC1 expression in DRG neurones. However, the inability of the TAC1 promoter to respond to MAPK activation prompted us to consider the possibility that a second regulatory sequence was involved in the regulation of TAC1 by the MAPK.

The spatially complex expression patterns displayed by many genes often rely on the presence of remote regulatory elements that modulate the activity of gene promoters in a tissue-specific or stimulus-inducible manner [47]. In addition, there is evidence that the interaction of these remote regulatory elements with the promoters of the genes they regulate is highly specific [48,49,50]. In the past, the identification of these remote regulatory elements by deletion analysis was very labour intensive and time consuming. However, identification of remote regulatory elements using comparative genomics has become possible recently following the sequencing of multiple vertebrate genomes [21,22,30,32,51,52]. We embarked on a comparative genomic analysis of the TAC1 locus over many hundreds of kilobases as the majority of the regulatory sequences required for TAC1 regulation lay within 380 kb of the TAC1 gene [53,54]. We succeeded in identifying a short but highly conserved sequence that allowed the TAC1 promoter to respond to MAPK activation and present further evidence that this induction depended on the MEK/ERK signalling pathway. These studies were only possible due to the availability of relatively efficient Amaxa- and magnetofection-based methods for transfecting plasmid DNA into primary DRG neurones.

The possibility of a mechanistic linkage of the ERK pathway with the regulation of TAC1 is consistent in the context of much of the current literature. For example, prior to injury or noxious stimuli, both ERK activity and TAC1 expression are largely confined to small diameter nociceptive C-fibre neurones [6,13]. However, an increase in ERK activation and an increase in the expression of TAC1 expression is associated with induced hyperalgesia [6,55]. Furthermore, ERK activation and the expression of TAC1 in larger diameter neurones is associated with noxious stimulation and hyperalgesia [6,19,56,57]. These studies were supported by the observation that, as for TAC1, the MEK/ERK pathway is upregulated by capsaicin and that ERK is essential to the regulation of nociceptor sensitivity by activation of TRPV1 [58]. Furthermore, genetic knockouts of TAC1, the production of dominant negative mouse mutants of the MEK gene or the application of an alternative MEK/ERK antagonist U0126 reversed the ability of noxious stimuli to cause hyperalgesia [3,4,5,14,59,60]. Thus, several lines of evidence have already suggested close parallels between ERK activation and TAC1 expression. In addition, a possible link to ERK activation and expression of SP was suggested when bradykinin-evoked release of SP from C-fibre neurones was reduced in the presence of MEK inhibitors [61]. Thus, the repression of the angiotensin-induced activity of ECR2-TAC1prom by the MEK antagonist described in the current study is entirely consistent with these previous observations. What was unexpected, however, was the requirement of long-distance regulatory synergy and selectivity between an enhancer and the TAC1 promoter in this MEK/ERK driven activation. This observation is unique and the ability to carry out these studies using long-range comparative genomics has great potential for the understanding of the regulation of many other genes involved in the disease process.

The significant upregulation of ECR2-TAC1-Luc activity observed following treatment with the antagonist SB202190 suggests that p38MAPK may play a role in antagonising the influence of MEK/ERK on the TAC1 gene. The maintenance of gene expression levels through regulatory homeostasis is a well-known phenomenon and, based on the current data, it is not entirely unexpected to find that the expression of the TAC1 gene is maintained in this way in sensory neurones [62,63,64,65].

In order to demonstrate the tissue-specific nature of ECR2-TAC1prom activity, we used transgenic mouse lines to demonstrate that ECR2 permits the tissue-specific activation of the TAC1 promoter in SP expressing cells of the DRG. SP expression is a well-characterised marker of sensory neurones, and our transgenic experiments demonstrate that the synergy between ECR2 and the TAC1 promoter is sensory neurone-specific within DRG. Thus, the significant levels of co-expression of the ECR2-TAC1prom-LacZ transgene with SP argues strongly for the involvement of ECR2/ TAC1 promoter synergy in the cell-specific expression of the TAC1 gene in sensory neurones. These transgenic experiments further support the relevance of ECR2 in the sensory neurone-specific expression of TAC1.

The inability of ECR2 to induce sensory neurone-specific activity of either the hβglobin promoter or the minimal TATA box promoter demonstrates the high degrees of enhancer-promoter specificity that is required to support the expression of the TAC1 gene in these cells. Although comparative genomics holds considerable promise for the identification of important long-range regulatory elements, its usefulness will be limited if enhancer-promoter specificity is not considered and the requirement for regulatory synergy in tissue-specific and inducible gene regulation is ignored. Therefore, we recommend that, wherever possible, the endogenous promoters of genes adjacent to candidate enhancer regions should be used to assay the properties of the candidate instead of exogenous promoters such as hβglobin or the heat-shock promoters commonly used.

We also wanted to determine whether the TAC1 promoter could respond to noxious stimuli. Capsaicin treatment has been used as an accepted paradigm for noxious stimulation and has also been widely used in the in vivo study of hyperalgesia [66,67]. Capsaicin has also been shown to induce expression of TAC1 in vivo [38,39] and gene deletions of the SP-receptor (NK1) significantly reduces the hyperalgesic affects of capsaicin [68]. We used DRG neurones transfected with either luciferase or LacZ reporter constructs to demonstrate that, in common with angiotensin, the TAC1 promoter requires ECR2 to respond to capsaicin. Another important observation was that capsaicin could upregulate the co-expression of both SP and the ECR2-TAC1prom-LacZ transgene in larger diameter cells in transgenic DRG explants. These observations support the hypothesis that synergy between the ECR2 and TAC1 promoter sequences are responsible for the expression of SP observed in larger diameter neurones that has previously been observed in inflammatory disease models and hyperalgesia [6,7,35,37,69]. We were also able to show that the effects of capsaicin on the activity of the ECR2-TAC1prom constructs were reliant on the activation of the MEK/ERK pathway in these neurones, strongly suggesting the involvement of this pathway in the induction of the TAC1 gene during noxious stimulation.

Progress in understanding the mechanisms governing gene regulation in the maintenance of health and the progression of disease has severely lagged behind our understanding of the roles of genes and proteins. This lag is understandable considering the difficulty in identifying the regulatory sequences responsible for controlling cell-specific gene regulation which, unlike coding sequences, have no predictable structure and may be located many tens or hundreds of kilobases from the genes that they regulate [51,52]. The current study demonstrates that many of the obstacles that prevented a detailed analysis of the regulation of genes involved in the inflammatory response can be overcome using comparative genomics, primary DRG neurone transfection and transgenic mouse analysis.
